# Multimodal prehabilitation to enhance functional capacity of patients with esophageal cancer during concurrent neoadjuvant chemotherapies—a randomized feasibility trial

**DOI:** 10.1093/dote/doae087

**Published:** 2024-10-08

**Authors:** Jade St-Pierre, Miquel Coca-Martinez, Kenneth Drummond, Enrico Minnella, Agnihotram V Ramanakumar, Lorenzo Ferri, Franco Carli, Celena Scheede-Bergdahl

**Affiliations:** Department of Kinesiology and Physical Education, McGill University, Montreal, Canada; Department of Anesthesia, McGill University Health Centre, Montreal, Canada; Department of Anesthesia, Maisonneuve-Rosemont Hospital, University of Montreal, Canada; Faculty of Medicine and Health Sciences, McGill University, Montreal, Canada; Faculty of Dental Medicine and Oral Health Sciences, McGill University, Montreal, Canada; Faculty of Medicine and Health Sciences, McGill University, Montreal, Canada; Research Institute, McGill University Health Center, Montreal, Canada; Division of Thoracic Surgery, Department of Surgery, McGill University Health Centre, Montreal, Canada; Department of Anesthesia, McGill University Health Centre, Montreal, Canada; Department of Kinesiology and Physical Education, McGill University, Montreal, Canada; Department of Anesthesia, McGill University Health Centre, Montreal, Canada

**Keywords:** esophageal neoplasms, feasibility studies, functional status, neoadjuvant therapy, pre-operative exercise

## Abstract

Esophageal adenocarcinoma continues to bear high morbidity and mortality. Prehabilitation, using exercise, nutrition, and psychosocial strategies to optimize patients prior to surgical resection, is largely underexplored in this malignancy, especially in patients undergoing neoadjuvant chemotherapy. Objectives of this study were (i) to determine feasibility of prehabilitation during treatment in patients with esophageal cancer and (ii) to establish differences between hospital and home-based exercise. Patients were recruited from August 2019 – February 2023 and blindly randomized to either supervised or homebased exercise, receiving identical nutritional and psychosocial support. The main outcome measures were recruitment, retention, and dropout rates. The secondary outcomes included cardiorespiratory fitness, functional capacity, and quality of life. Forty-four subjects were blindly randomized: 23 to supervised exercise and 21 to home-based exercise (72% recruitment rate). Overall compliance for the supervised group was 72%; home-based group was 77%. Baseline to pre-operative, both groups experienced significant increases in sit-to-stand, arm curls, and amount of weekly moderate–vigorous physical activity. The home-based group experienced an additional considerable decrease in up-and-go test times. Both groups maintained cardiorespiratory fitness and saw substantial increases in some quality-of-life scores. Multimodal prehabilitation is feasible for patients with esophageal cancer undergoing neoadjuvant chemotherapy. In both groups, patient fitness, which is relevant for this patient population given the anticipated decline in functional status during this period, was maintained. This study provides a foundation for future prehabilitation interventions in this patient population.

## Graphical Abstract

**Graphical Abstract:**
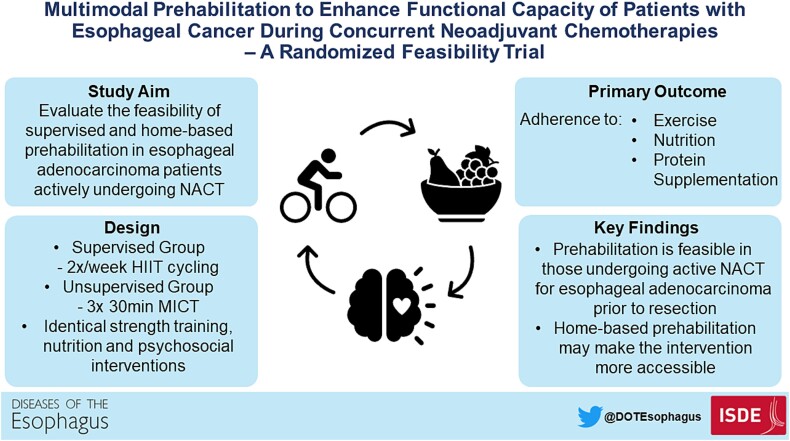

## INTRODUCTION

Esophageal cancer is currently the sixth leading cause of cancer-related death and seventh most frequently diagnosed worldwide.^1^ Unfortunately, due to lack of early symptom awareness and widespread screening tool, the disease is often diagnosed at an advanced stage and accompanied by a high physical burden of illness. Standard of care in Canada for esophageal adenocarcinoma (EAC) dictates neoadjuvant chemotherapy (NACT) followed by resection and subsequent adjuvant chemotherapy (ACT).^2^ Difficulty swallowing or dysphagia, a hallmark symptom of the disease, is highly distressing and contributes to a compromised nutritional state, pain, and rapid deterioration of quality of life (QoL).^3^ NACT, although intended for treatment, can further diminish patient functional capacity, induce toxicity, and can decrease time-to-treatment-failure at the cost of modestly-increased survival.^4^ With the combination of disease-related physiological decline, compounded by chemotherapy-derived toxicity, there is the potential for the EAC patient to be both physically and mentally ill-prepared for the stress of upcoming surgery.

A proposed intervention to mitigate these declines in patient function and QoL is multimodal prehabilitation which combines exercise, nutrition, and psychosocial support to optimize patients for surgery.^5^ Given the standard wait time of roughly 3 months between diagnosis and surgery due to NACT in the EAC treatment plan, it is an ideal opportunity for prehabilitation to be implemented. This is especially important as patients often undergo ACT after invasive resection, possibly further benefiting from optimization before the added toll of subsequent cytotoxic therapy.

Prehabilitation in patients actively undergoing NACT is understudied. The often-deteriorated state of patients undergoing these therapies causes a hesitancy to prescribe exercise during this period. Therefore, data from a feasibility trial will provide important insight into what is reasonable for this patient population. Additionally, many prehabilitation studies utilize a supervised format where patients are expected to travel to the study site or gym facility for exercise training. While supervision is often preferred for research and clinical care, it is resource-intensive and can be out of reach for those living in rural communities^6^ as well as those negatively impacted by cancer treatment and its side effects.^7^ In recent years, and with the impact of the COVID-19 pandemic, there has been increased interest in home-based training.^8^ As this study’s tertiary care hospital center serves a large geographical area, not all patients are realistically able or willing to attend supervised training. In a landscape of largely heterogeneous prehabilitation interventions, this study will be exploratory in nature and lay the foundation for what can be tolerated by this specific patient population.

The objectives of the study were two-fold: (i) to determine feasibility, as determined by recruitment rate, dropout rate, and compliance to components of the prehabilitation intervention in patients with esophageal cancer undergoing NACT and (ii) to determine if home-based, unsupervised exercise provides comparable benefits to hospital-based, supervised exercise in terms of cardiorespiratory fitness, functional capacity, and QoL.

## METHODS

Patients were assessed for eligibility at their initial visit to the Upper Gastrointestinal Clinic at the McGill University Health Center (MUHC) from 2019–2023. Eligibility for participation in the study included aged 18 years or older and referred for elective management of non-metastatic esophagogastric cancer, with planned NACT followed by surgery. Exclusion criteria included comorbid medical, physical, and mental conditions that contraindicate exercise or oral nutrition including acute or unstable cardiac conditions, disabling orthopedic and neuromuscular disease, or presence of feeding gastrostomy/jejunostomy. Patients with poor English or French comprehension were also excluded, as were patients residing >50 km from the MUHC due to blind randomization, possibly introducing transportation limitations if in the supervised group.

### Trial design

This study was a parallel arm, randomized, single-blind study conducted at the MUHC (Montreal, Quebec, Canada). The trial protocol was approved by the MUHC Research Ethics Board (ESOPH-Prehab/2019-5387), and written informed consent was obtained from each patient before randomization. This study recruited from August 2019 to February 2023.

### Sample size calculation

Data suggests that, in patients with esophageal cancer undergoing NACT, V̇O_2_AT decreases by 2.19 mL kg(−1) min(−1) (95% confidence interval [CI] 1.47–2.91).^9^ To detect this difference between groups, 19 subjects per arm are required to reach a statistical power of 80%, and a type I error of 0.05. As our previous trial reported a 17% loss to follow-up,^10^ a total of 54 participants were required.

### Study design

Eligible patients were randomized in a 1:1 ratio to either supervised or a home-based exercise prehabilitation group. Participants were randomized using computer-generated blocks of six, and group assignments were placed in sequentially numbered opaque envelopes. Due to the nature of the intervention, it was impossible to blind participants or health care professionals to group allocation.

### Statistical analysis

The normality of data was assessed both graphically and using the Kolmogorov–Smirnov and Shapiro–Wilk normality tests. Continuous variables were presented depending on the nature of distribution; means, standard deviations (SD) for normally distributed data, and medians and interquartile ranges for non-normally distributed data. Between-group comparisons of continuous variables was performed using independent-samples *t*-tests or analysis of variance, while changed in continuous variables over time were evaluated using paired *t*-tests or Wilcoxon tests. Categorical variables were compared using the chi-squared or Fisher’s exact tests. Statistical analysis was performed using SPSS version 26 (IBM, New York, USA).

### Outcomes

The primary outcome in this study is feasibility as determined by (i) recruitment rate, (ii) dropout rate, and (iii) compliance to components of the prehabilitation intervention (exercise, nutritional advice, and protein supplementation).

Secondary outcomes include three subcategories: cardiorespiratory fitness, functional capacity, and QoL. Within cardiorespiratory fitness, measures of interest include anaerobic threshold (VO_2_AT), an objective value of oxygen uptake at which anaerobic metabolism occurs and commonly associated with poor perioperative outcomes for patients after major surgery.^11^ VO_2_AT was measured by a cardio-pulmonary exercise test (CPET) as per the Perioperative Exercise Testing and Training Society practice guidelines. All tests were analyzed by an anesthesiologist accredited in CPET assessment.

Functional capacity was measured by a 6-minute walking test (6MWT), as per American Thoracic Society recommendations, sit-to-stand (STS), timed-up-and-go (TUG), and left and right arm curls. The Duke Activity Status Index (DASI) and Community Healthy Activities Model Program for Seniors (CHAMPS) questionnaire were used to measure functional capacity and frequency of physical activities, respectively.

QoL is essential in this patient group, as the arduous treatment plan can have serious negative effects.^12^ This study used the Edmonton Symptom Assessment (ESAS) questionnaire, which rates the intensity of common symptoms, and the Functional Assessment of Cancer Therapy—Esophagus (FACT-E), which is composed of the FACT-General with the addition of an esophageal subscale. Finally, the Hospital Anxiety and Depression Screening (HADS) questionnaire was included in patient assessment so that it could be determined whether the patient displays symptoms of anxiety, depression, or a combination of both.

#### Prehabilitation intervention

Patients were approached in the Upper GI clinic and provided with information about the study. If interested, they were invited to a baseline appointment within two weeks of the initial meeting. At this visit, all patients signed an informed consent form and underwent tests evaluating fitness including walking, strength, and aerobic capacity as outlined in the secondary outcomes. After evaluation, patients were blindly randomized to either the supervised, hospital-based exercise or the unsupervised, home-based exercise group. The supervised group was required to travel to the clinic twice per week for one-on-one training with the kinesiologist where they performed high-intensity interval training (HIIT) and resistance training. The home-based group was prescribed an aerobic exercise protocol^10^ and the same resistance training program as the supervised group. Patients continued their training during chemotherapy and up until the day of surgery. All patients, no matter the exercise allocation, underwent NACT during training. The majority received mDCF + avelumab (40%) or FLOT (50%) protocols with 31/38 patients completing their prescribed NACT regimens (i.e. 100% adherence).

##### Aerobic exercise program—supervised (hospital-based)

The aerobic component included HIIT, consisting of high-intensity bouts of exercise interspersed with active recovery periods.^13^ Using the baseline CPET-derived variables, an individualized protocol was created for each participant. High-intensity bouts, 4 × 3-minutes each, were conducted at 85%–90% of workload at peak oxygen consumption (peak V̇O_2_), and recovery bouts, 4 × 4-minute intervals, were performed at 80%–85% of workload at oxygen consumption at anaerobic threshold (V̇O_2_AT). However, given that patients often felt ill due to NACT, an adapted supervision protocol was developed. High-intensity bouts, 4 × 3-minutes, were conducted at a target heart rate of 80%–95% of the predicted peak heart rate or a self-reported intensity of 14–17 (6–20 Borg rating of perceived exertion scale, or RPE) and recovery bouts, 4 × 4-minute interval, were conducted at 40%–50% of predicted peak heart rate or an RPE of <9.

##### Aerobic exercise program—unsupervised (home-based)

The home-based program included continuous moderate-intensity aerobic exercise three times a week for 30 minutes each session. Exercise modalities included brisk walking, jogging, or cycling, depending on personal preference. Patients were instructed to self-monitor intensity to reach 12–13 on the 6–20 Borg rating of perceived exertion scale.

##### Resistance training—both groups

Resistance training included 8 exercises targeting major muscle groups (upper limb, trunk, lower limb), performed for 2–3 sets of 8–12 repetitions. The intensity was based on initial strength capacity, using elastic bands (Thera-Band®, Akron, OH, USA). The kinesiologist selected the resistance level to reach moderate-intensity effort, rated as 5–6 on a 10-point scale. Supervised participants trained with a kinesiologist, while home-based participants were provided with logbooks to record all activities and monitored via weekly telephone calls by the kinesiologist.

## COVID-19 ADDENDUM

As COVID-19 pandemic struck during this research study, a Zoom option for supervised training was developed should patients be unable or unwilling to come to the hospital in person. The Zoom.us application version 5.13.0 (San Jose, USA) is recognized to respect Public Health Information Protection Act. A cycling ergometer was delivered to the patient’s home and a secure video conference link was used to connect. The exercise sessions were conducted via Zoom with direct supervision by the kinesiologist. Of note, five patients utilized this Zoom option for training, with the remaining choosing to continue in person.

### Nutritional therapy

All patients received nutritional optimization. At baseline, participants completed a 3-day estimated food record. A dietitian assessed dietary habits and anthropometric data to create a comprehensive status evaluation and to calculate the required amount and relative proportion of macronutrients. The metabolic requirement was adjusted to meet the increased nutritional demand for their upcoming surgery and food-based dietary advice was provided.

### Protein supplementation

Whey protein supplement protein powder (Enhanced Medical Nutrition, Toronto, Ontario) was prescribed to guarantee a daily protein intake of 1.2–1.5 g/kg of ideal body weight.

### Psychosocial interventions

All patients participated in at least one session with a trained nurse. Strategies taught included relaxation techniques, mind-framing, and deep breathing. As participation was not required for this study, adherence was not recorded.

### Perioperative care

The ERAS protocol for esophagectomy has been implemented at our tertiary care center since 2008. As such, all patients received standardized perioperative care.

## RESULTS

### Primary outcomes

**Fig. 1:**
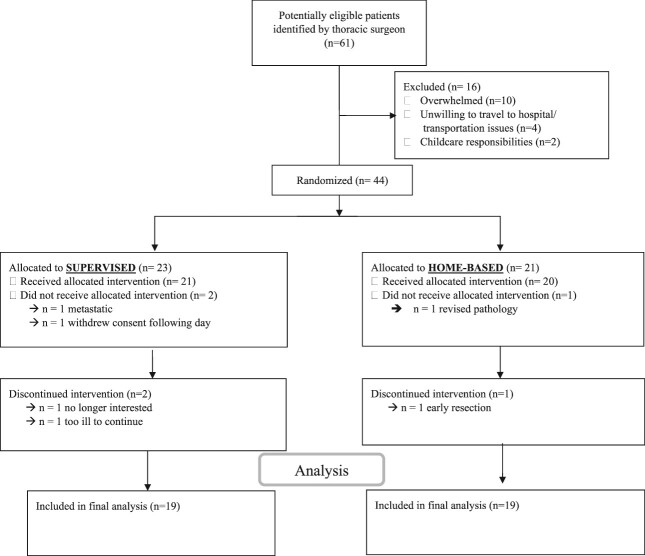
Study CONSORT diagram.

A total of 61 patients were identified as candidates by the treating thoracic surgeon (timeframe: August 2019 and February 2023). Recruitment was paused due to COVID-19 restrictions in place from March to September 2020. Sixteen patients decline participation, with the most common reasons being not interested or overwhelmed (*n* = 10), not willing to travel to the hospital/transportation issues (*n* = 4), childcare responsibilities/commitments (*n* = 2), and COVID-related clinic closure prior to baseline evaluations (*n* = 1). Forty-four patients provided informed consent (Fig. 1). One patient withdrew consent the following day so did not undergo baseline assessment. Forty-three patients underwent baseline assessment (recruitment rate: 72%). Demographics and baseline data are presented in Table 1. Of those who agreed to participate, one was excluded due to metastasis, one had a revised treatment plan, and three withdrew (one patient felt too ill to continue, one was no longer interested, and one had early emergency resection). Recruitment was terminated once 19 subjects per arm were reached as per the power calculations previously described.

**Table 1:** Patient baseline characteristics

Demographics	Supervised (*n* = 22)	Unsupervised (*n* = 21)	*P*-value
Age, yrs	65 [7.5]	64 [9.5]	0.53
Gender, male	15 (68%)	17 (81%)	0.33
BMI, kg/m^2^	28.18 [5.33]	27.62 [5.74]	0.60
Lean body mass %	53.25 [13.29]	55.03 [11.73]	0.88
Frailty (median)	2 (1)	1 (2)	0.53
PG-SGA	12 [5]	10 [5]	0.31
LOS	12 [14]	9 [8]	0.04
Comorbidities			
Diabetes	7 (32%)	6 (29%)	0.81
Hypertension	10 (45%)	10 (48%)	0.88
CVD	3 (14%)	2 (10%)	0.67
Dyslipidemia	9 (41%)	12 (57%)	0.44
GERD	13 (59%)	10 (48%)	0.55
Level of tumor			
Thoracic middle 1/3	0	1	
Thoracic lower 1/3	9	5	
GEJ Siewart 1	3	6	
GEJ Siewart 2	5	5	
GEJ Siewart 3	5	4	
Procedure			
Ivor Lewis	13	18	
3 hole	4	1	
Transabdominal	0	1	
Left thoracoabdominal	4	0	
Left thoracoabd ext-tot	1	0	
Other	0	1	
ASA			
II	14	13	
III	8	7	
IV	0	1	
Dysphagia			
0	4	4	
1	6	11	
2	5	2	
3	7	4	

Overall mean compliance for the supervised group was 72% (aerobic training: 59%; resistance: 60%; nutrition: 91%; supplementation: 76%). Meanwhile, the home-based group had an overall adherence of 77% (aerobic training: 78%; resistance: 68%, nutrition: 85%; supplementation: 81%). Mean length of the intervention was 107 days (SD 28).

### Secondary outcomes

Mean increase in VO_2_AT from baseline to pre-operative was 0.52 mL/min/kg in the supervised group and 0.09 mL/min/kg in the home-based group. Results are presented in Tables 2 and 3.

**Table 2:** Supervised exercise group—baseline to pre-operative functional and aerobic capacity, QoL

Time point	Baseline	Pre-operative		
	Mean (SD)	Mean (SD)	Mean difference from BL	*P*-value
Functional and aerobic capacity				
6MWT	480 [120]	490 [131]	+ 10	0.59
STS	12 (4)	14 (4)	+ 2	0.006
TUG	7.24 (1.84)	6.26 (1.89)	−0.98	0.041
Right arm curl	16 (5)	19 (5)	+ 3	0.009
Left arm curl	14 (5)	19 (4)	+ 5	0.001
VO2-AT	11.41 (2.36)	11.93 (3.397)	+ 0.52	0.73
DASI	42.2 (15.07)	45.5 (14.47)	+ 3.3	0.57
CHAMPS total	96 [141]	136 [187]	+ 40	0.19
Mod + vig	36 [93]	71 [122]	+ 35	0.003
QoL				
HADS (total)	11 (6)	9 (5)	−2	0.20
ESAS	21 [15]	18 [14]	−3	0.49
FACT-E total	107 [31]	138 [23]	+31	0.001
Physical	21 [6]	23 [5]	+ 2	0.58
Social	20 [5]	24 [4]	+ 4	0.03
Emotional	15 [3]	18 [4]	+ 3	0.009
Functional	15 [4]	20 [4]	+ 5	0.001
Esophageal	42 [10]	55 [12]	+ 13	0.001
TOI	78 [16]	97 [17]	+ 19	0.001

**Table 3:** Home-based exercise group—baseline to pre-operative functional and aerobic capacity, QoL

Time point	Baseline	PREOP		
	Mean (SD)	Mean (SD)	Mean difference from BL	*P*-value
Functional and aerobic capacity				
6MWT	521 [97]	510 [112]	−11	0.92
STS	13 [3]	15 [3]	+ 2	0.03
TUG	6.78 [1.44]	6.57 [1.53]	−0.21	0.63
Right arm curl	18 [5]	21 [4]	+ 3	0.01
Left arm curl	18 [5]	20 [4]	+ 2	0.03
VO2-AT	11.94 [2.098]	12.03 [2.706]	+ 0.09	0.97
DASI	45.1 [16]	45.7 [16.29]	+ 0.6	0.88
CHAMPS total	42 [25]	84 [45]	+ 42	0.19
Mod + vig	14 [11]	44 [33]	+ 30	<0.001
QoL				
HADS (total)	13 (6)	11 (6)	−2	0.51
ESAS	21 (11)	16 (13)	−5	0.22
FACT-E total	115 (28)	131 (25)	+ 16	0.19
Physical	22 [6]	22 [6]	+ 0	0.91
Social	23 [3]	24 [2]	+ 1	0.76
Emotional	14 [7]	17 [5]	+ 3	0.27
Functional	16 [7]	16 [7]	+ 0	0.72
Esophageal	40 [14]	53 [12]	+ 13	0.02
TOI	70 [33]	83 [32]	+ 13	0.27

Both groups improved their sit-to-stand (supervised +2 repetitions, unsupervised +2 repetitions) and both arm curls values (unsupervised +2/+2 repetitions, supervised +3/+5 repetitions) while the supervised group also demonstrated lower TUG times (−1 second). There were no differences in DASI scores. Both groups experienced increases in performed moderate and vigorous physical activity, unsupervised from 14–44 minutes and supervised from 36–71 minutes, as measured by the CHAMPS questionnaire.

Both groups saw increases in QoL scores as measured by the FACT-E esophageal sub-score (unsupervised +13, supervised +13). The supervised group also increased total (+31), social (+4), and emotional (+3) FACT-E sub-scores. The TOI (trial outcome index), which combines physical, functional, and emotional scores also increased (+19) in the supervised group. As for the ESAS questionnaire, a decrease for the home-based group (−4.9) and supervised group (−2.77) was observed. Generally, the minimally clinically important difference (MCID) for this questionnaire is 1 point.^14^

## DISCUSSION

This study demonstrates that both supervised hospital-based and unsupervised home-based prehabilitation programs are feasible for patients with esophageal cancer undergoing NACT. Preliminary exploration of secondary outcomes revealed that both exercise allocations provided similar benefits: there was overall maintenance of cardiorespiratory fitness and significant improvement of functional capacity and QoL, in line with a previous UK study.^15^ Considering the usual physical and mental decline previously reported to occur in this timeframe,^16^ maintenance of these patient parameters is important and should not be underestimated. In addition, being able to offer prehabilitation programs to suit a variety of patient needs and health care resources allows for more inclusive patient optimization and an important degree of flexibility.

### Explanation of findings

Overall adherence to supervised prehabilitation was 72%. Meanwhile, the home-based group had a general adherence of 77%, above the 70% generally accepted for home-based interventions.^17^ While there is no agreed-upon benchmark for acceptability in exercise research adherence,^18^ adherence for supervised exercise interventions in other thoracic cancers ranges from 72%–88% attendance rate.^19^^,^^20^ The adherence to the home-based exercise component was higher than the supervised (78% aerobic/68% resistance vs 59% aerobic/60% resistance, respectively), understandably as the nature of supervised prehabilitation trainings can present barriers including transportation, finding the time to participate^21^ and displacement to location of exercise training.^22^ Leaving home to participate in exercise programs may discourage patients from attending should they feel ill due to treatment side effects.^7^^,^^23^ Conversely, home-based participants have the flexibility in their exercise schedule, especially for those who experience nausea, diarrhea, or physical discomforts.^7^

### Secondary outcomes

Patients maintained their cardiorespiratory fitness, overall functional capacity and reported an improved QoL. Although there was no increase in AT, it is important that this parameter was maintained throughout the study as a direct association exists between preoperative CPET values and postoperative outcomes in patients undergoing cancer surgery.^24^ Previous work has shown that patients, left without an intervention whilst undergoing chemotherapy for esophageal cancer, typically demonstrate a deterioration in CPET values, specifically VO_2_AT;^9^^,^^25^^,^^26^ therefore, mitigating this decline is a triumph in this population.

There was no difference in 6MWT which is surprising given our center’s past research.^10^ However, both groups’ baseline walking distance was high (480 supervised, 521 unsupervised), still well >400 m, a prognostic cut-off frequently used in other populations.^27^ This maintained walking distance indicates that patients are in approximately the same condition as baseline, despite several rounds of cytotoxic therapies. Both groups saw significant increases in sit-stand and bicep curls. The supervised group demonstrated an additional considerable decrease in TUG. These changes are likely attributable to the muscular strength program, which remained identical between groups, except for the supervision component. While the sample size is small, these modest increases are particularly important for this group as decline is common during this difficult treatment period.^28^ Expectedly, the CHAMPS moderate + vigorous score increased, as a direct result of the planned exercise intervention.

Given the elevated morbidity and mortality of esophageal cancer, it could be argued that health-related QOL is as important an endpoint as survival, if not more so.^29^ The ESAS, which rates the intensity of common symptoms, decreased by 4.93 points for the home group and by 2.77 points for the supervised group. Generally, the MCID is 1 point.^14^ Both groups saw a statistically significant increase in the esophageal subscale score which asks about disease-specific symptoms. This is consistent with NACT as most patients have an important decrease or near complete resolution of dysphagia during treatment.^30^ However, overall, the supervised group experienced more statistically significant increases in the subcategories of the FACT-E, including the total score, functional and emotional sub-score, and TOI score, obtained from 3 of the FACT-E subscales: physical well-being, functional well-being, and additional concerns (esophageal).

Limitations of this study include lack of a true control group and the study not being a full randomized control trial (RCT). Given that our main objective was to investigate the feasibility of two potential programs and not the magnitude of responses to prehabilitation itself, we believed that a control group would not serve the purpose of the study. Additionally, recruiting true ‘sedentary’ individuals is becoming an increasingly difficult task. As knowledge of ‘exercise is good’ is becoming increasingly common in the preoperative space (e.g. booklets and pre-surgical clinics), one cannot be confident that control patients undertake no physical activity after diagnosis. As for the study not being a full RCT, we opted for a more explorative approach, considering the limited information pertaining to this population in the current literature. While this type of investigation does not provide powerful statistical results or conclusions, it serves as a steppingstone as prehabilitation continues to expand in this population.

Strengths of our research include using a multimodal strategy, encouraging holistic treatment of patients, personalizing exercise based on individual CPET test values and a goal of addressing flexibility in programming. Reflecting a more patient-centered approach in modern healthcare and a need to create relevant programs, prehabilitation must take into consideration what each patient wants and needs during this period of their cancer care trajectory. No matter how medically sound a prescription may be, if the patient does not follow the program, compliance and subsequent benefits will not occur.

## CONCLUSIONS

Prehabilitation is feasible in patients with esophageal cancer undergoing NACT and that program flexibility is possible. This feasibility trial can inform future large RCTs and provide options and possible expected benefits of prehabilitation in this patient population.
